# Genomic, Transcriptomic, and Epigenomic Features Differentiate Genes That Are Relevant for Muscular Polyunsaturated Fatty Acids in the Common Carp

**DOI:** 10.3389/fgene.2019.00217

**Published:** 2019-03-15

**Authors:** Hanyuan Zhang, Peng Xu, Yanliang Jiang, Zixia Zhao, Jianxin Feng, Ruyu Tai, Chuanju Dong, Jian Xu

**Affiliations:** ^1^Key Laboratory of Aquatic Genomics, Ministry of Agriculture, CAFS Key Laboratory of Aquatic Genomics and Beijing Key Laboratory of Fishery Biotechnology, Chinese Academy of Fishery Sciences, Beijing, China; ^2^Fujian Collaborative Innovation Center for Exploitation and Utilization of Marine Biological Resources, Xiamen University, Xiamen, China; ^3^Henan Academy of Fishery Science, Zhengzhou, China; ^4^College of Fishery, Henan Normal University, Xinxiang, China

**Keywords:** common carp, polyunsaturated fatty acids, GWAS, transcriptome, methylation

## Abstract

Polyunsaturated fatty acids (PUFAs) are a set of important nutrients that mainly include arachidonic acid (ARA4), docosahexaenoic acid (DHA), eicosapentaenoic acid (EPA), and α-linolenic acid (ALA). Recently, fish-derived PUFAs have been associated with cardiovascular health, fetal development, and improvement of brain functions. Studies have shown that fish muscular tissues are rich in PUFAs, which are influenced by various factors, including genetic variations, regulatory profiles, and methylation status of desaturase genes during fatty acid desaturation and elongation processes. However, the genetic mechanism and the pathways involved in fatty acid metabolism in fishes remain unclear. The overall aim of this study was to assess differences in gene expression responses among fishes with different fatty acid levels. To achieve this goal, we conducted genome-wide association analysis (GWAS) using a 250K SNP array in a population of 203 samples of common carp (*Cyprinus carpio*) and identified nine SNPs and 15 genes associated with muscular PUFA content. Then, RNA-Seq and whole genome bisulfite sequencing (WGBS) of different groups with high and low EPA, DHA, ARA4, and ALA contents in muscle, liver and brain tissues were conducted, resulting in 6,750 differentially expressed genes and 5,631 genes with differentially methylated promoters. Gene ontology and KEGG pathway enrichment analyses of RNA-Seq and WGBS results identified enriched pathways for fatty acid metabolism, which included the adipocytokine signaling pathway, ARA4 and linoleic acid metabolism pathway, and insulin signaling pathway. Integrated analysis indicated significant correlations between gene expression and methylation status among groups with high and low PUFA contents in muscular tissues. Taken together, these multi-level results uncovered candidate genes and pathways that are associated with fatty acid metabolism and paved the way for further genomic selection and carp breeding for PUFA traits.

## Introduction

Aquatic products contribute to a large part of our daily diet, which not only offer high-quality protein but also contain abundant long-chain polyunsaturated fatty acids (PUFA), vitamins, and mineral substances. The fat contents (FAs) of most fishes range from 1 to 4% (Iverson et al., 2002). In general, fish flesh has lower FA but higher PUFA content compared to livestock and poultry meat (Rymer and Givens, 2005; Wood et al., 2008; Henriques et al., 2014; Geay et al., 2015). Omega-6 and omega-3 PUFAs are essential fatty acids (EFAs) that play critical roles in maintaining cell membrane structure. However, as bioactive lipid mediators, these are not synthesized by the human body (Calder, 2013). Long-chain PUFAs, particularly eicosapentaenoic acid (EPA) and docosahexaenoic acid (DHA), positively influence retinal development, neurological development, and cardiovascular system maintenance (Innis, 2008; Guesnet and Alessandri, 2011; Mozaffarian and Wu, 2011). PUFAs can effectively reduce the risk of heart diseases (e.g., coronary heart disease and heart rate variability) (Vedtofte et al., 2011; Enns et al., 2014), prevent the increase of blood viscosity (Zanetti et al., 2015), and act as an anti-glioma agent that promotes the regression and apoptosis of tumors by acting as an intracellular signaling molecule (Witte and Hardman, 2015). Another two PUFAs, namely, α-linolenic acid (ALA) and arachidonic acid (ARA4), have also been reported to be beneficial for human development and health by preventing cardiovascular disease and promoting embryonic and brain development (Davis-Bruno and Tassinari, 2011; Pan et al., 2012).

Fatty acids in fishes originate from any of two sources, namely, synthesis *in vivo* from non-lipid carbon sources or uptake from dietary lipids. Therefore, muscular PUFA content can be influenced by dietary intake and endogenous metabolism (Davidson, 2013). Various studies have investigated the mechanism underlying PUFA synthesis and degradation, including associated genetic variants, gene expression, and epigenetics (Berton et al., 2016; Chen et al., 2018). The pathways for the synthesis and modification of PUFAs include a variety of enzyme systems. Genetic variants in fatty acid-synthesizing enzymes substantially influence muscular fatty acid levels (Foster, 2012; Beld et al., 2015). For example, delta-5 desaturase (FADS1) and delta-6 desaturase (FADS2) are two enzymes involved in fatty acid metabolism (Ralston et al., 2015). Two haplotypes including the *fads1* and *fads2* genes have been associated with differences in the synthesis of long-chain PUFAs (Ameur et al., 2012). During fatty acid synthesis, acetyl-CoA serves as the initial specific substrate for the acetyl-CoA carboxylase. Modifications in the microsomal glycerol-3-phosphate pathway in fishes as well as in mammals involve the incorporation of PUFAs into phospholipids and triacylglycerols (Murphy, 2013). During desaturation and elongation processes in freshwater fishes, omega-9 fatty acids could be synthesized by Δ9 desaturase, whereas omega-3 (n-3) and omega-6 (n-6) fatty acids could not be produced due to the lack of the Δ12 and Δ15 desaturase enzymes.

Although PUFAs have a series of important functions, current understanding of the molecular mechanisms related to fish fatty acid metabolism remains limited. QTL mapping and genome-wide association analysis (GWAS) on muscular FA have been reported in the Atlantic salmon, common carp (*Cyprinus carpio*), and large yellow croaker (Derayat et al., 2007; Sodeland et al., 2013; Kuang et al., 2015; Xiao et al., 2016; Zheng et al., 2016). A previous genome-wide scan identified multiple QTLs located on LG6 and LG23 that influence n-3 PUFA content in the muscle tissues of Asian seabass (Xia et al., 2014). Fatty acid desaturases and elongases (e.g., Δ5 and Δ6 desaturases) of several freshwater and marine fishes have been cloned (Agaba et al., 2004; Zheng et al., 2016). These enzymes play crucial roles in the biosynthesis of the long-chain C20/22 PUFAs from shorter chain C18 PUFAs. It involves a series of complex biosynthesis reactions, including desaturation, elongation and peroxisomal β-oxidation, to convert ALA to n-3 fatty acids EPA and DHA (Kjaer et al., 2016). A previous study demonstrated that there are two alternative pathways for DHA biosynthesis in teleost, (Oboh et al., 2017). One is Sprecher pathway which desaturates 24:5(n-3) to 24:6(n-3) via Δ6 desaturase Fads2, and the other is Δ4 pathway functioned by Δ4 desaturase Fads2. However, the molecular genetic mechanisms of fatty acid-related traits, particularly those involved in important PUFA (e.g., DHA, EPA) pathways, remain unclear.

The common carp has a long breeding history as an important worldwide cultured fish (Bostock et al., 2010). In 2016, the global production of *C. carpio* reached 4.56 million tons, while 3.50 million tons were cultured in China^1^. Dozens of *C. carpio* strains and populations have been cultured, including Yellow River carp, Songpu mirror carp, and Hebao carp. The diverse populations of *C. carpio* exhibited phenotypic variations in body color, growth, scales, and muscular fatty acid contents. The muscular PUFA content of common carp have been a research topic of interest based on the need to increase aquaculture quality instead of quantity. Based on its economic and scientific importance, genomic resources of *C. carpio* have been developed and extensively utilized in recent years. The transcriptome and genome assembly of *C. carpio* have been reported, paving the way for more in-depth investigations (Ji et al., 2012; Xu J. et al., 2012, Xu P. et al., 2014). A high-throughput carp SNP array with 250K SNPs was developed, thereby offering a powerful tool for genetic association studies (Xu J. et al., 2014). Several QTL and GWAS of *C. carpio* were conducted using the Carp 250K SNP array, including traits of growth and FA (Peng et al., 2016; Zheng et al., 2016). High-throughput sequencing techniques facilitated the multi-omics studies on genome resequencing, genome methylation, and transcriptome analysis on important traits of *C. carpio* (Jiang et al., 2014; Wang et al., 2014a,b). Gene expression profiles are largely regulated by DNA methylation through transcriptional regulation and chromatin remodeling. The genomic regions with differential methylation levels, known as differentially methylated regions (DMRs), represent the most active regions that may be related to transcriptional regulation. A handful of integrated studies on the expression and epigenetics of various species have been reported. He et al. (2013) investigated transcriptomic and epigenomic variations in maize hybrids and concluded that similar mechanisms may account for the genome-wide epigenetic regulation of gene activity and transposon stability in different organs. Zhang et al. (2015) conducted integrative analysis of transcriptomic and epigenomic data to reveal regulatory patterns for bone mineral density variations, showing consistent association evidence from both mRNA/miRNA expression and methylation data. A recent study conducted comparative transcriptomic and DNA methylation analyses of color trait in Crucian carp and identified several pigmentation-related pathways (Zhang et al., 2017). The above studies indicated the power of multi-omics data analysis. Here, we report our multi-level research on muscle PUFA content traits in common carp using GWAS, RNA-Seq, and methylation analyses. Dozens of fatty acid metabolism-associated candidate genes and pathways were identified by the integration of associated genes, differentially expressed genes (DEGs), and genes in differential methylated regions (DMRs). This study takes a further step to better understand the mechanism of muscular PUFA content and presents potential applications in breeding.

## Materials and Methods

### Ethics Statement

This study was conducted in accordance with the recommendations of the Care and Use of Animals for Scientific Purposes established by the Animal Care and Use Committee of the Chinese Academy of Fishery Sciences (ACUC-CAFS). The protocol was approved by the ACUC-CAFS. Before the blood and tissue samples were collected, all fishes were euthanized in MS222 solution.

### Sample Collection and Phenotypic Measurements

A total of 203 Yellow River 2-year-old carp were randomly selected from a large population that was cultured at the Henan Fishery Research Institute, Henan, China. From each sample, 1 mL of blood was collected in lysis buffer for DNA extraction and genotyping. More than 100 g of dorsal muscle tissue were collected and cryopreserved in dry ice, and then stored at −80°C until fatty acid content determination. More than 5 g of muscle, liver, and brain tissues were acquired and preserved in RNALater (Qiagen, Hilden, Germany) at −80°C for RNA extraction and sequencing. Approximately 20 g of muscle sample per fish were used in measuring total fat and fatty acid contents (*N* = 12), which were conducted by the Agricultural Products Safety and Quality Supervision Inspection Center in Zhengzhou, Henan according to national standards for food safety. Correlation analysis among traits was conducted using IBM SPSS Statistics 19.0 software, and the correlation matrix plot was drawn using the R package ggplot2^2^.

### DNA Extraction, Genotyping, and Quality Control

Genomic DNA was extracted from whole blood using a DNeasy 96 Blood & Tissue Kit (Qiagen, Shanghai, China) following the manufacturer’s protocol. The extracted DNA was quantified using a NanoDrop-1000 spectrophotometer (Thermo Fisher Scientific, Wilmington, DE, United States). The integrity of DNA was examined on a 1.5% agarose gel by electrophoresis. The final DNA concentration was diluted to 50 ng/μL for genotyping. The total amount of qualified genomic DNA for whole genome sequencing was 2 μg per sample. The common carp 250K SNP array was developed in a previous study using the Affymetrix Axiom genotyping technology (Xu J. et al., 2014). Genotyping was performed by GeneSeek (Lincoln, Nebraska, United States). After genotyping, PLINK v1.9^3^ was used for quality control (Chang et al., 2015). SNPs with low call rate (<95%) or low minor allele frequency (MAF < 5%) were excluded, and samples with <90% genotyping rates were filtered out.

### Genome-Wide Association Analysis

Genome-wide association analysis for screening of fat and fatty acid content trait (total fat, 12 individual fatty acids, nine classified fatty acids)-related genes were performed based on genotyping data using the TASSEL version 5.0 software^4^ (Bradbury et al., 2007). The model “PCA” was used to create a q-matrix, and then the option “wMLM” was used to perform association analysis. The genome-wide significant *P*-value threshold was adjusted based on Bonferroni correction, and the suggestive threshold was set to 3.445 × 10^−5^. Associated SNP loci for four PUFA (ALA, ARA4, DHA, and EPA) content values were selected with a corrected *P* value <0.05. Gene annotation was performed on 10 kb of the flanking regions of the associated SNP loci. The Manhattan plots and Q-Q plots were generated using qqman package of the Comprehensive R Archive Network^5^.

### RNA Sequencing and Differential Gene Expression Analysis

Three tissues (brain, liver, and muscle) of 20 fishes were included in RNA sequencing based on four traits, namely, ALA, ARA4, DHA, and EPA content. From these fishes, four were selected with high content and four were selected with low content in all four traits. Total RNA was extracted from the brain, liver, and muscle tissues using an RNeasy kit (Qiagen, Shanghai, China) following manufacturer’s instructions. The integrity and size distribution of all samples were assessed using a Bioanalyzer 2100 system (Agilent Technologies, Santa Clara, CA, United States). A complementary DNA (cDNA) library was constructed, and high-throughput sequencing was performed using an Illumina HiSeq2500 Sequencing System with paired-end 2 × 150 nucleotide reads (Illumina, San Diego, CA, United States).

Low-quality reads and residual adapter sequences from FASTQ files were filtered and trimmed using Trimmomatic v.0.32 (Bolger et al., 2014). Reads were trimmed when the average Phred score was <20 across four bases in the sliding window. After filtering, reads shorter than 36 bp or single ends were removed. Bowtie2-build indexer (Bowtie2 v.2.3.4.2) (Langmead and Salzberg, 2012) was used to build a Bowtie index from the common carp genome assembly. The filtered reads were aligned to the genome sequence using Tophat2 (Kim et al., 2013). Samtools (Li et al., 2009) was used to index the Tophat2 output bam files, and Cufflinks (Trapnell et al., 2010, 2014) was used to assemble the reconstructed transcripts from the aligned reads using genome and annotation files. These assembled transcript structures were merged into one single dataset with Cuffmerge. Differential expression and regulation at the gene and transcript levels were identified using Cuffdiff. The volcano plots showing gene expression differences were constructed using the ggplot2 package. The Benjamini-Hochberg FDR corrected *P* value (*q* value) <0.05 and the absolute log_2_ fold-change (FC) value >1 were considered differentially expressed genes. The heatmaps of the four PUFAs in three tissues were generated by the FPKM of differentially expressed genes using the pheatmap package. Gene Ontology (GO) enrichment and Kyoto Encyclopedia of Genes and Genomes (KEGG) enrichment analyses were performed using DAVID Bioinformatics Resources 6.8 Tools^6^ (Huang et al., 2009). Pathways with at least five differentially expressed genes assigned and *q* value <0.05 were considered enriched. The GO and KEGG plots were constructed using R package clusterProfiler (Yu et al., 2012).

### Whole Genome Bisulfite Sequencing and Differential Methylation Analysis

Genomic DNA was extracted from the muscle tissues of the same fishes used in the RNA-Seq study. Genomic DNA was treated using sodium bisulfite, which converts unmethylated cytosine to uracil, then thymine (Wang et al., 2006). Whole genome bisulfite sequencing (WGBS) was performed using an Illumina HiSeq2500 sequencer with 150-bp paired-end sequencing (Illumina, San Diego, CA, United States). The software swDMR^7^ was used to comprehensively analyze the DMRs from methylation sequencing profiles by a sliding window approach (Wang et al., 2015). The input files were prepared using a WGBS data aligner Bismark (Krueger and Andrews, 2011). The DMR detection and annotation procedures were performed as described below. First, a sliding window with 1,000-bp window size and 100-bp step size was chosen for scanning methylation rates. Second, The Benjamini-Hochberg FDR corrected *P* value (*q* value) <0.1 and absolute log_2_ FC value >1 were considered as potential DMRs. Third, two potential DMRs were merged when their distance was less than the threshold. The merged DMRs were tested by previous steps to guarantee the significance level. This extension step was repeated until *P* value >0.1. The new extension of potential DMRs were considered as candidate DMRs. Finally, candidate DMRs in the promoter regions were selected for enrichment and correlation analyses. GO and KEGG enrichment analyses were performed using DAVID Bioinformatics Resources 6.8 Tools^6^ (Huang et al., 2009). The GO and KEGG plots for candidate genes in the DMPs were constructed using R package clusterProfiler (Yu et al., 2012). The DGE and DMP results were correlated using R package ggplot2 (Ginestet, 2011), and transcriptional factor binding site (TFBS) prediction was conducted using AnimalTFDB 3.0 Tools^8^ (Hu et al., 2018).

## Results

### Genotyping and Phenotyping of 203 Accessions of *C. carpio*

On the basis of our previous work (Xu J. et al., 2014; Xu P. et al., 2014), we randomly collected 203 samples from a cultivated population of *C. carpio*. After DNA extraction and SNP genotyping using Carp 250K SNP array, a raw genotype data of 250,000 SNPs for 203 samples were generated. A total of 193 samples with 108,684 polymorphic SNPs passed the quality control threshold, and 29,026 tag SNPs were chosen by the pruning method (SNPs with LD R^2^> 0.9 were filtered out) for further association analysis.

**FIGURE 1:**
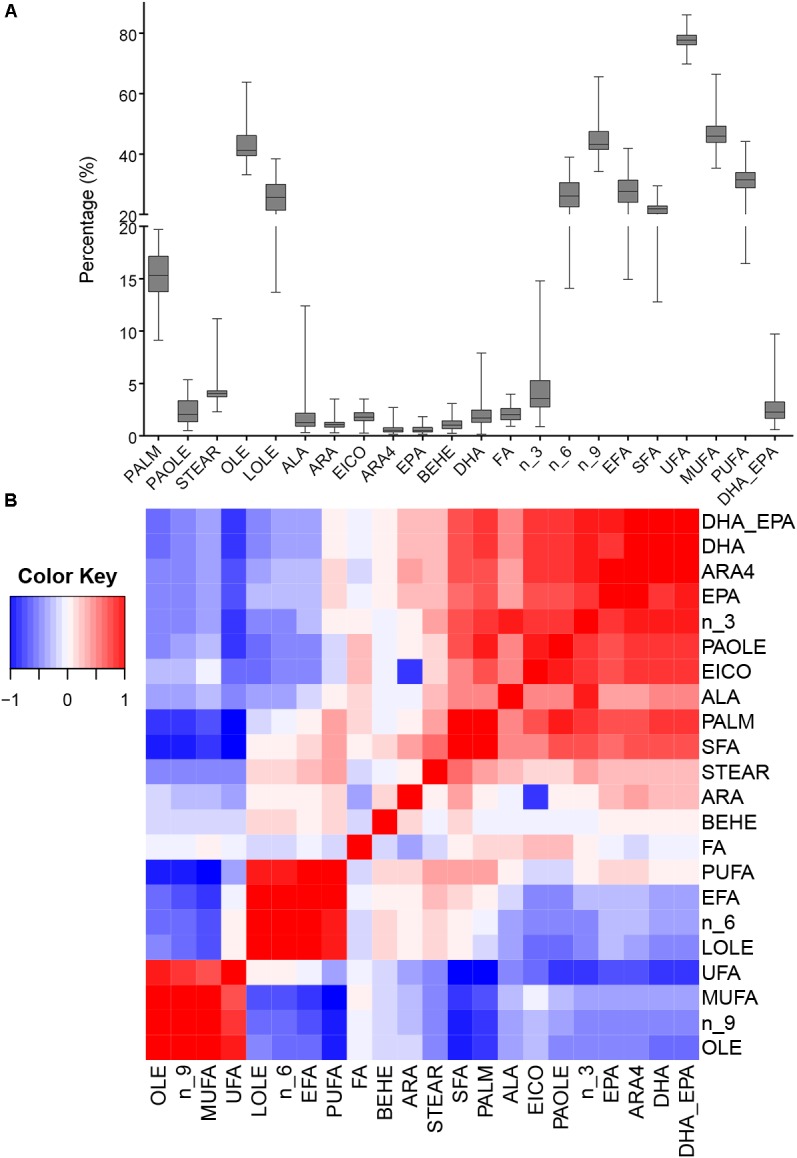
Phenotypic distribution and correlation heatmap. **(A)** Box plot of phenotype distribution. The X-axis represents phenotypes, and the Y-axis represents the proportion in the total fatty acids. **(B)** Correlation of all phenotypes. The colors represent the correlation coefficient R^2^ values: red means positive correlation and blue means negative correlation.

We analyzed total FA and 12 fatty acids, then nine classified groups of fatty acids were also calculated (Table 1). Figure 1A shows that oleic acid and linoleic acid comprise 60% of all fatty acids, and the content of the PUFAs (ALA, ARA4, DHA, and EPA) are relatively low. The 22 phenotypes were also analyzed to uncover potential relationships among phenotypes, and the correlation matrix is shown in Figure 1B and Supplementary Table S1. We found that ALA, ARA4, DHA and EPA contents were significantly correlated with each other, possibly suggesting shared or related genes regulating these traits. The correlations between ALA and other three fatty acids (ARA4, DHA, EPA) were moderate (*r* = 0.398, 0.434, 0.306, respectively; *p* < 10^−8^, *p* < 10^−9^, *p* < 10^−4^, respectively), whereas the measurements of ARA4, DHA, and EPA were highly correlated (ARA4 vs. DHA: *r* = 0.916, *p* < 10^−76^; ARA4 vs. EPA: *r* = 0.705, *p* < 10^−29^; DHA V.S. EPA: *r* = 0.684, *p* < 10^−27^).

**Table 1:** Summary of fat, nine classified fatty acids, and 12 single fatty acids.

Number	ID	Terminology	Fatty acids included/Abbreviation
1	FA	Fat content	–
2	n_3	omega 3	ALA, EPA, DHA
3	n_6	omega 6	LOLE, ARA4
4	n_9	omega 9	OLE, EICO
5	SFA	saturated fatty acid	LOLE, ALA
6	EFA	essential fatty acid	PALM, STEAR, ARA, BEHE
7	UFA	unsaturated fatty acid	PAOLE, OLE, LOLE, ALA, EICO, EPA, DHA
8	MUFA	monounsaturated fatty acid	PAOLE, OLE, EICO
9	PUFA	polyunsaturated fatty acid	LOLE, ALA, EPA, DHA
10	DHA_EPA	highly unsaturated fatty acid	DHA, EPA
11	PALM	palmitic acid	C16:0
12	PAOLE	palmitoleic acid	C16:1
13	STEAR	stearic acid	C18:0
14	OLE	oleic acid	C18:1n9c
15	LOLE	linoleic acid	C18:2n6c
16	ALA	α-linolenic acid	C18:3n3
17	ARA	arachic acid	C20:0
18	EICO	eicosaenoic acid	C20:1n9
19	ARA4	arachidonic acid	C20:4n6
20	EPA	eicosapentaenoic acid	C20:5n3
21	BEHE	behenic acid	C22:0
22	DHA	docosahexenoic acid	C22:6n3

### Genome-Wide Association Analysis and Gene Annotation of Identified SNPs

**FIGURE 2:**
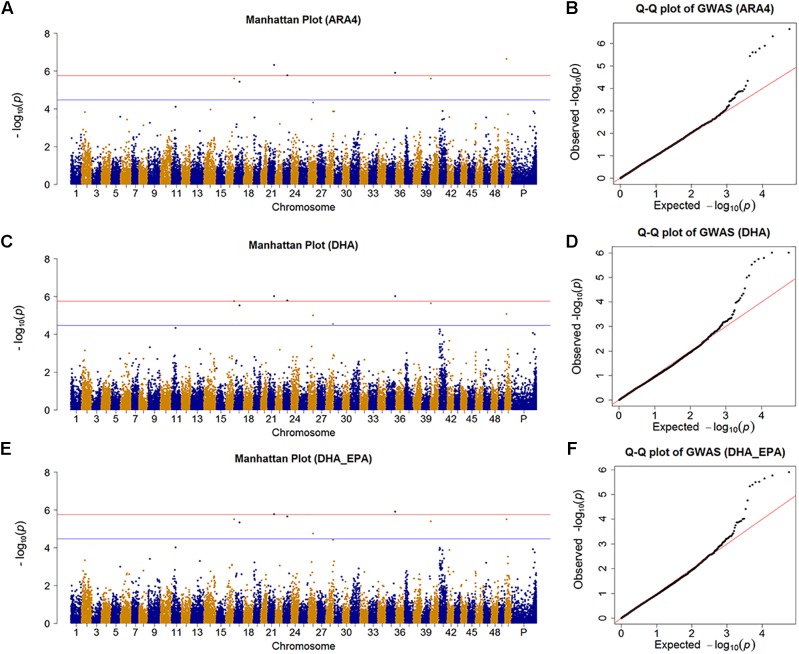
Manhattan plot and Q-Q plot for traits with significant SNPs. **(A)** Manhattan plot for the GWAS results of muscular ARA4 content. The red line indicates the significant threshold while the blue line shows the suggestive threshold. **(B)** Q-Q plot for GWAS results of muscular ARA4 content. **(C)** Manhattan plot for the GWAS results of muscular DHA content. **(D)** Q-Q plot for the GWAS results of muscular DHA content. **(E)** Manhattan plot for the GWAS results of muscular DHA + EPA content. **(F)** Q-Q plot for GWAS results of muscular DHA + EPA content.

We identified nine SNPs (7, 8, 2, and 1 SNP for ARA4, DHA, EPA, and ALA, respectively) that achieved the suggestive significance threshold (*P* < 3.445 × 10^−5^), in which 4 SNPs (4, 3, 0, and 0 SNPs for ARA4, DHA, EPA, and ALA, respectively) surpassed the significance line (*P* < 1.72 × 10^−6^) for the content of four fatty acids. To further identify more potentially associated SNPs, we further investigated the results of the DHA_EPA group, which contained two associated SNPs and six suggestive SNPs (Figure 2 and Supplementary Table S2). The Manhattan plots and Q-Q plots are shown in Figure 2 for traits with associated SNPs (ARA4, DHA, and DHA_EPA), in which the Q-Q plots (genomic inflation factor ≈1) indicated reliability of experimental design and data analysis. Genes within the 10-kb regions of the associated and suggestive SNPs were annotated using the common carp genome (Xu P. et al., 2014), and a total of 15 genes were identified (six genes for associated SNPs, 12 genes for suggestive SNPs).

### Transcriptomic Profiling of Samples With Divergent PUFA Contents

We assessed the fatty acid content of 20 new individuals and selected eight showing extreme PUFA content (H: high content group; L: low content group). The ARA4, EPA, DHA, and ALA content of the 20 individuals are shown in Supplementary Table S3. Three types of tissues (brain, liver, and muscle) were collected from eight individuals, and we used Illumina high-throughput sequencing to generate mRNA transcriptomes. The overall quality of the extracted RNA from 24 tissue samples is summarized in Supplementary Table S4. After sequencing, approximately 176 Gb of raw data were generated from 24 tissue samples, and 173.1 Gb clean data were used for further differential gene expression (DGE) analysis (Supplementary Table S5). Using the Bowtie software, 76.1% of the clean reads could be mapped to the genome, and the DGE of each trait between the high-content and low-content groups was calculated using the Cufflinks software.

**FIGURE 3:**
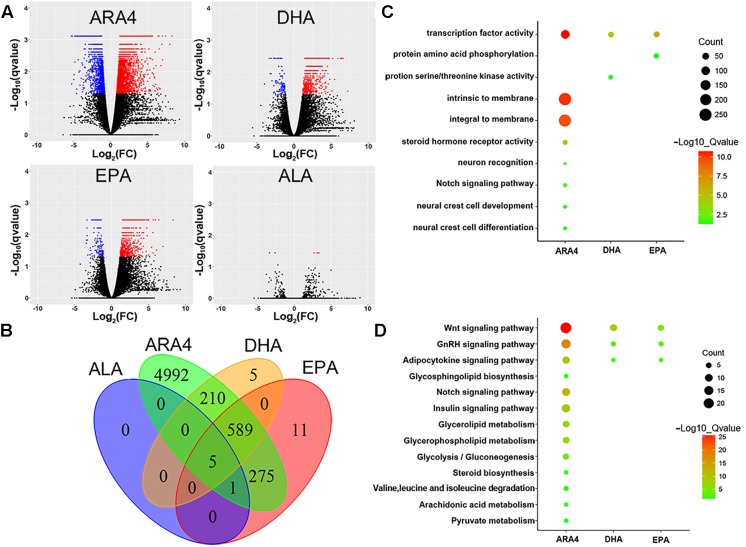
Volcano plot, Venn diagram, Gene Ontology, and KEGG enrichment for differentially expressed genes in brain tissues. **(A)** Volcano plots for DEGs in the brain tissues for four traits. Red dots indicate upregulated genes; blue dots indicate downregulated genes. **(B)** Venn diagram of DEGs in brain tissues showing the number of shared and unique genes for each trait. **(C)** Gene Ontology enrichment of DEGs in brain tissues. The size of the circles represents gene numbers in each term; colors represent minus logarithms of adjusted *P* values. **(D)** KEGG enrichment of DEGs in brain tissue. Size of circles represents gene numbers in each pathway; colors represent minus logarithms of adjusted *P* values.

As shown in the volcano plots in Figure 3, a large number of genes were identified to be differentially expressed in the brain, with counts of 6,191 genes, 916 genes, 1,010 genes, and 36 genes for ARA4, DHA, EPA, and ALA, respectively (Figure 3 and Supplementary Table S6). We further conducted GO and KEGG enrichment analyses to focus on the most important pathways (Figure 3 and Supplementary Tables S6–S8). Several important lipid and fatty acid metabolism pathways were enriched, including “adipocytokine signaling pathway,” “ARA4 metabolism,” and “glycerolipid metabolism.” Key genes within these enriched pathways included *cd36*, *npy*, and *lepr*. Interestingly, several development and metabolism-related pathways were also enriched (Figure 3C,D) such as “Wnt signaling pathway,” “Notch signaling pathway,” and the “insulin signaling pathway,” indicating possible relationship between growth and muscular fatty acid. Pathways of amino acid metabolism (especially branched chain amino acids) were also enriched such as “valine, leucine, and isoleucine degradation,” indicating related pathways between amino acid and fatty acid metabolism processes.

In liver tissues, 113, 299, 228, and 60 genes were identified to be differentially regulated in the four groups, respectively (Supplementary Figure S1 and Supplementary Tables S9–S11). Similar to the results of the brain tissues, pathways related to growth and amino acid metabolism were also enriched in the liver tissues such as the “insulin signaling pathway,” “glycine, serine, and threonine metabolism,” and the “Wnt signaling pathway,” including key genes such as *prkaa2* and *gsk-3*β. In the muscles, there were 23, 15, 38, and 4 genes that were differentially expressed in the four groups, respectively (Supplementary Figure S2 and Supplementary Table S12). Volcano plots and Venn diagrams showed a limited number of genes that were differentially expressed in the muscle tissues, thus no GO terms or KEGG pathways were enriched. Supplementary Figure S3 shows the results of cluster analysis of the four traits in three types of tissues.

### Patterns of Methylation Variations Among Groups With Distinct PUFA Contents

**FIGURE 4:**
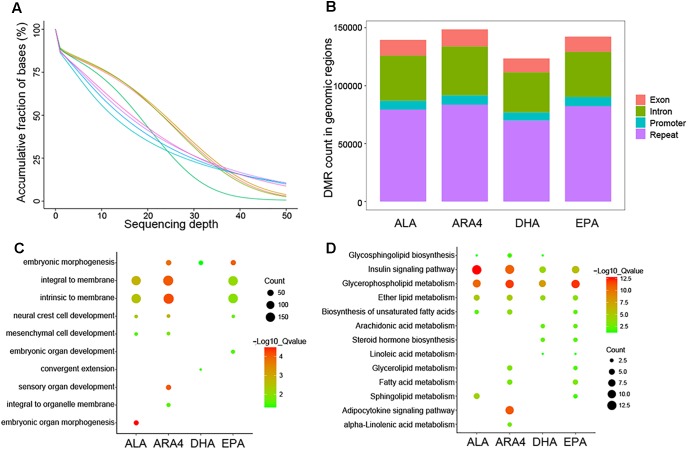
Sequencing depth distribution, DMR classification, and enrichment of genes within DMP regions. **(A)** Accumulative fraction against sequencing depth. Curve lines with different colors represent eight samples used for WGBS, and the Y-axis represents the cumulative ratio of genomic regions mapped by reads. **(B)** Counts for each DMR classification in different genomic regions for each trait. **(C)** Gene Ontology enrichment of genes downstream of DMPs. The size of the circles represents the number of genes in each term; colors represent minus logarithms of adjusted *P* values. **(D)** KEGG enrichment of genes downstream of DMPs. The size of the circles represents the number of genes in each pathway; colors represent minus logarithms of adjusted *P* values.

To assess the global trends of epigenetic variations in different groups, we performed genome-wide pairwise comparisons of each epigenetic modification between the H and L groups. Muscle tissues were collected from the eight samples used in transcriptome analysis, and DNA was extracted for further WGBS. A total of 541.3 Gb of raw data (3,608,912,206 raw reads) were acquired (around 45× coverage for each sample), and 535.1 Gb of clean data were used for alignment to the carp genome (Figure 4A and Supplementary Table S13). Following standard pipelines (see Methods), DMRs were identified (Figure 4B). The most abundant DMRs were located in intergenic repeat regions, followed by introns, exons, and promoter regions (Supplementary Table S14). Because DMRs in the promoter regions (differentially methylated promoters, DMPs) play vital roles in the regulation of transcription (Haque et al., 2016), we selected DMPs for further functional enrichment analysis (Supplementary Tables S15, S16). GO enrichment analysis identified various terms, including “embryonic organ development,” “embryonic organ morphogenesis,” which were related to growth and development (Figure 4C). More direct evidences have been identified in KEGG enrichment such as shared pathways (Figure 4D), namely, “insulin signaling pathway,” “glycerophospholipid metabolism,” “ether lipid metabolism” for the four traits, and fatty acid metabolism-related pathways, including “ARA4 metabolism,” “linoleic acid metabolism,” “biosynthesis of unsaturated fatty acids,” and “adipocytokine signaling pathway.” Furthermore, “steroid hormone biosynthesis” was enriched, indicating the role of fatty acids in hormone metabolism.

### Integrated Analysis of DGE and DMP Results

**FIGURE 5:**
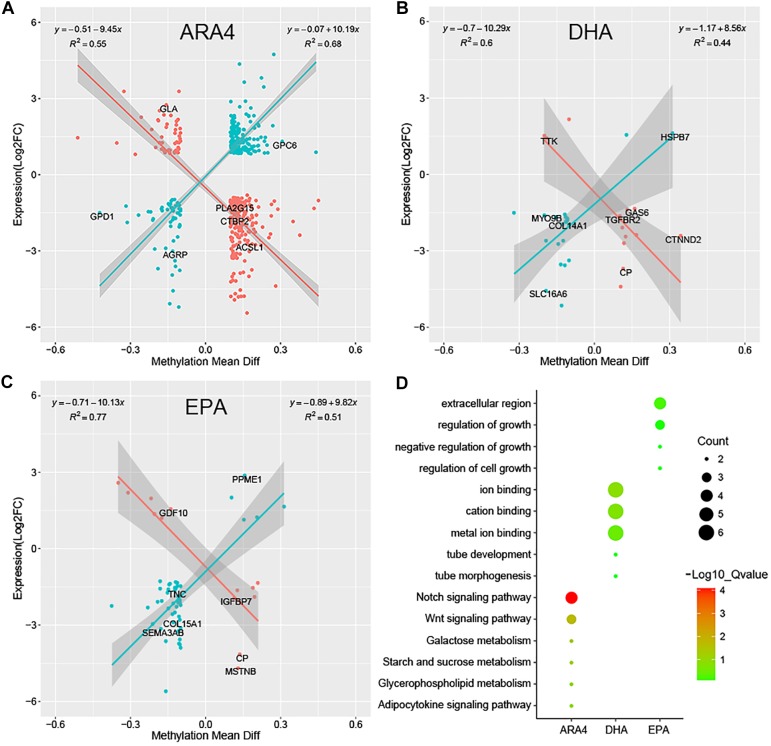
Pearson correlation of gene expression fold changes and differential methylation rates for DEGs in brain tissues and enrichment analysis. Scatter plots for **(A)** ARA4, **(B)** DHA, and **(C)** EPA. The X-axis represents mean difference in methylation ratio between high and low ARA4 content groups; the Y-axis represents the logarithm of fold changes (log_2_FC). The red dots represent negatively correlated genes and blue dots represent positively correlated genes. The trend lines and formula in each scatter plot represent the correlation coefficients. **(D)** GO and KEGG enrichment of correlated genes for three traits. The size of circles represents the number of genes in each pathway; colors represent minus logarithms of adjusted *P* values.

Although DGE and DMP analyses identified several genes and pathways, the correlation between the two regulatory levels remains unclear. Methylation levels in the promoter regions significantly affect the transcription of downstream genes; therefore, it is necessary to conduct a correlation analysis of genes shared between the DGE and DMP results. Due to the limited number of genes shared between the liver/muscle DGE and DMP results, we focused on the correlation between brain DGE log_2_(FC) values and DMP gene methylation differences (Supplementary Table S17). Figure 5 shows the results of Pearson correlation analysis, which indicated a significant linear correlation for the ARA4, DHA, and EPA traits. The genes shown in Figure 5 were classified into two categories, namely, positively correlated (blue dots) and negatively correlated (red dots). For the ARA4, DHA, and EPA traits, 457, 35, and 59 genes were included in the correlation analysis. The R^2^ values of the 221 positively correlated genes and 236 negatively correlated genes in ARA4 trait analysis were 0.68 and 0.55, respectively. For the DHA trait, the R^2^ values of 21 positively correlated genes and 14 negatively correlated genes were 0.44 and 0.60, respectively. For the EPA trait, R^2^ values of 46 positively correlated genes and 13 negatively correlated genes were 0.51 and 0.77, respectively. The DGE and DMP results of the liver and muscle tissues were also integrated (Supplementary Table S17), which identified fewer genes compared to those in the brain. To find more evidence supporting the correlation between gene expression and genome methylation, TFBS prediction of the promoters of selected genes from integrated analyses was performed (Supplementary Table S18).

## Discussion

### GWAS of 203 Individuals and Gene Annotation

Among the identified SNPs in GWAS, the most significant SNP carp222748 has been associated with ARA4 content in *C. carpio*, which is located within the coding region of the *duox2* gene. The Duox2 (dual oxidase 2) protein, which is encoded by *duox2*, generates hydrogen peroxide, which is required for the activity of thyroid peroxidase and plays a role in thyroid hormone synthesis. Interestingly, another gene, *trh* (pro-thyrotropin-releasing hormone), was identified downstream of a suggestive SNP carp215797, and the Trh protein stimulates the release of thyrotropin. Thyroid hormone status affects metabolic pathways of ARA4 in mice and human (Fonseca et al., 2014; Yao et al., 2015), and thus it is possible that a similar genetic mechanism may be utilized in ARA4 metabolism in *C. carpio*. Another associated SNP carp152877 was found to be associated with the ARA4, DHA, and DHA_EPA traits and is located downstream of the *ormdl3* gene. The Ormdl3 (ORM1-like protein 3) protein negatively regulates sphingolipid synthesis and may be indirectly involved in endoplasmic reticulum-mediated calcium ion signaling (Breslow et al., 2010). As the genes discovered from GWAS analysis may not be enough to illustrate the underlying mechanism of muscular PUFA metabolism, we sought to uncover more evidences by investigating gene expression and epigenetic statuses of the collected samples.

### GO and KEGG Analysis Based on PUFA RNA-Seq Data

GO and KEGG enrichment analyses of the DGE results identified a handful of genes that are involved in enriched pathways that are related to fatty acid metabolism. CD36 has been reported as a high affinity receptor for long-chain fatty acid (FA) uptake, in addition to the contribution of lipid accumulation and FA-initiated signaling (Pepino et al., 2014). Neuropeptide Y (NPY), an orexigenic hypothalamic neuropeptide that is released by the neurons of arcuate nuclei, influences foraging behavior and food intake in mammals and indirectly affects fat accumulation (Levin et al., 2013). Leptin is an adipocytokine that regulates energy intake and expenditure through interactions with the leptin receptor (LEPR). LEPR has been associated with intramuscular fat and FA accumulation in Duroc pigs (Ros-Freixedes et al., 2016). We presume these genes may have similar functions in *C. carpio*. Insulin has been reported to promote development and fatty acid biosynthesis through the conversion of glucose into triglyceride in the liver, fat, and muscle cells (Laron, 2008; Xu X. et al., 2012; Pramfalk et al., 2016).

Among the potentially associated genes identified in liver tissue pathways, protein kinase α2 (PRKAA2) participates in lipid metabolism and energy homeostasis. A recent study on mice showed that lipopolysaccharide could significantly inhibit PRKAA2 expression (Tzanavari et al., 2016). In the Wnt and insulin signaling pathways, *gsk-3*β has been enriched in the liver tissues for DHA and EPA. GSK-3β acts as an upstream regulator of the ACSL family and lipid accumulation in hepatocytes (Chang et al., 2011). Previous studies have shown that high levels of amino acids upregulate hepatic fatty acid biosynthetic gene expression in trout hepatocytes (Dai et al., 2015). Despite intragroup variations in each group due to limited samples, distinct expression patterns were observed between the H (high) and L (low) groups. Interestingly, several genes identified in GWAS were also differentially expressed in three tissues, including *ormdl3*, *trh*, and *nptn*. This provided potential correlations between SNP frequency and relative gene expression. Despite the sample size of GWAS and RNA-Seq were both too small to make a comprehensive exploration of all trait-related genes, the results were still very informative for further larger sample analysis.

### Predicting Fatty Acid Metabolism-Related Genes With DNA Methylation Data

The core genes within DMP-enriched pathways included *gpd1, acsl1, fads2, elovl6*, and the *pla2* family (*pla2g15, pla2g12b*, and *pla2g6*), some of which have already been reported to be involved in PUFA metabolism in various species. For example, acyl-coenzyme A (CoA) syntheses 1 (ACSL1) is a well-studied obesogenic gene that is involved in fatty acid metabolism. The expression of ACSL1 has been reported to be associated with high caloric food intake in mice and human (Joseph et al., 2015). In the biosynthesis of PUFA, Δ6 desaturase (FADS2) has been demonstrated as an important indicator that catalyzes the first denaturation step and influences PUFA synthesis capability in fish. The expression of *fads2* is regulated by dietary fatty acid profiles in Japanese seabass and is significantly negatively correlated with CpG methylation rates in the *fads2* gene promoter (Xu H. et al., 2014). The elongation of very long chain fatty acid 6 (ELOVL6) mainly catalyzes the elongation of long chain SFAs and MUFAs (C14, C16, and C18), while it is engaged in balancing the overall fatty acid composition in mammals (Corominas et al., 2015).

### Integrated Analysis of DGE and DMP Results

Taken together, the significant linear correlation between expression and methylation indicates the epigenetic effects on transcription in addition to genetic factors such as genome variations. GO and KEGG enrichment further confirmed the importance of these correlated genes, and vital pathways were enriched, including “adipocytokine signaling pathway,” “glycerophospholipid metabolism,” “Notch signaling pathway,” “Wnt signaling pathway,” “ion binding,” and “regulation of growth”. Genes enriching these pathways included *agrp, ctbp2*, and previously discussed *acsl1, gpd1*, and *pla2g15*. AGRP has been reported to play similar roles in food intake and high-fat diet preference as NPY in mice and bats, and the upregulation of the *agrp* gene enhances diet preference and fat gain (Levin et al., 2013). The *ctbp2* gene was downregulated and highly methylated in the high-PUFA content group, which agrees with the findings of previous studies that overexpression of C-terminal-binding protein 2 (CTBP2) suppresses lipid accumulation and hepatic glucose uptake (Liu et al., 2017). Fewer correlated genes were identified from the liver and muscle data, partly because of the limited number of samples. The *acsl5* gene showed higher expression levels and lower methylation levels in the promoter region in the H group compared to the L group in relation to muscular ALA content. ACSL5 has been reported to act like other ACSL family members and engages in lipid biosynthesis and fatty acid degradation (Bowman et al., 2016). Besides, Fad and Elovl enzymes encoded by *fad* and *elovl* genes have been demonstrated to have all the activities and jointly play an important role for the biosynthesis of DHA from C_18_ PUFA in rabbitfish, as well as in other teleost (Monroig et al., 2012). In mammal and teleost fish, Elovl2, Elovl4, and Elovl5 have been cloned and functionally characterized separately as crucial elongation enzymes in PUFA biosynthesis (Agaba et al., 2005; Jakobsson et al., 2006; Morais et al., 2009; Monroig et al., 2010). It has been reported that the capability of PUFA biosynthesis varies among teleost fish with alternative pathways during evolution (Castro et al., 2016). Therefore, we assume *elovl6* which has been enriched in DMP pathway has similar functions in its encoding Eolvl6 enzyme. Fads2 with Δ4, Δ6, and Δ8 desaturase activities, respectively, composed a crucial part of PUFA biosynthesis as well (Li et al., 2010; Oboh et al., 2017). After integration of three types of analyses, the important genes identified in this study were summarized in Table 2, which provided a full view of genes supported by multiple evidences. We used muscles for WGBS and methylation analysis and considered that it could represent genome methylation of an individual. However, we could not ignore the slight differences in methylation among tissues. Investigations on tissue-specific methylation sites using multi-omics analyses and the correlation between DGE and DMP in the same tissue are thus warranted. Our results indicate that fatty acid metabolism-related genes are associated with the growth, hormone, and even amino acid relevant genes, which regulate muscular PUFA content.

**Table 2:** Summary of genes identified in three types of analyses.

Gene	Protein	Trait	Evidence
*duox2*	Dual oxidase 2	ARA4	GWAS
*trh*	Pro-thyrotropin-releasing hormone	ARA4, DHA	GWAS, RNA-Seq
*ormdl3*	ORM1-like protein 3	ARA4, DHA	GWAS, RNA-Seq
*lepr*	Leptin receptor	ARA4, DHA, EPA	RNA-Seq
*npy*	Neuropeptide Y	ARA4, DHA, EPA	RNA-Seq
*prkaa2*	Protein kinase α2	ARA4, DHA, EPA	RNA-Seq, Methylation
*gsk-3β*	Glycogen synthase kinase 3	DHA, EPA	RNA-Seq
*acsl1*	Acyl-coenzyme A (CoA) syntheses 1	ARA4	RNA-Seq, Methylation
*fads2*	Fatty acid desaturase 2	ARA4	Methylation
*elovl6*	Elongation of very long chain fatty acid 6	ARA4	Methylation
*agrp*	Agouti-related protein	ARA4	RNA-Seq, Methylation
*ctbp2*	C-terminal-binding protein 2	ARA4	RNA-Seq, Methylation
*acsl5*	Acyl-coenzyme A (CoA) syntheses 5	ARA4, ALA	RNA-Seq, Methylation

## Conclusion

This study investigated the associated genes and the divergence of transcriptomic and epigenomic variations in *C. carpio* samples with distinct muscular PUFA contents. The phenotypic correlation and GWAS results indicated dozens of potential genes that are associated with PUFA metabolism. Further investigation of DGE patterns of three tissues and gene profiles with DMPs identified important genes that enriched PUFA synthesis- and degradation-related pathways. Integrated analysis showed significant correlations between DGE and DMP and enrichment of correlated genes involved in vital pathways related to fatty acid desaturation, elongation, and transportation. Confirmation of these results using integrated genomic, transcriptomic, and epigenomic profiling with more extensive sequencing of larger samples is warranted. This study may also facilitate the applications of combined genome selection and molecular-associated breeding for multiple traits.

## Data Availability

The sequencing datasets of all samples have been deposited at NCBI (PRJNA493161).

## Author Contributions

JX initiated and coordinated the research project. HZ and JX conceived and conducted the analysis and drafted the manuscript. PX engaged in sample collection and genotyping analysis. YJ and ZZ assisted in transcriptome and methylation data analysis. JF, RT, and CD took part in trait measurement, tissue manipulation, and enrichment analysis. All the authors read and approved the final manuscript.

## Conflict of Interest Statement

The authors declare that the research was conducted in the absence of any commercial or financial relationships that could be construed as a potential conflict of interest.

## Footnotes

1 http://www.fao.org

2 https://cran.r-project.org/web/packages/ggplot2/

3 https://www.cog-genomics.org/plink2

4 http://www.maizegenetics.net/tassel

5 http://cran.r-project.org/package=qqman

6 https://david.ncifcrf.gov/

7 http://sourceforge.net/projects/swdmr/

8 http://bioinfo.life.hust.edu.cn/AnimalTFDB/#!/tfbs_predict
