# Supplementary material for: Genomic, Transcriptomic, and Epigenomic Features Differentiate Genes That Are Relevant for Muscular Polyunsaturated Fatty Acids in the Common Carp
Source: Front Genet. 2019 Mar 15;10:217. doi: 10.3389/fgene.2019.00217 (PMC6428711; doi:10.3389/fgene.2019.00217)

Supplementary Figure 1. Volcano plots, Venn diagram, and enrichment bubble plots for DEGs in the liver.

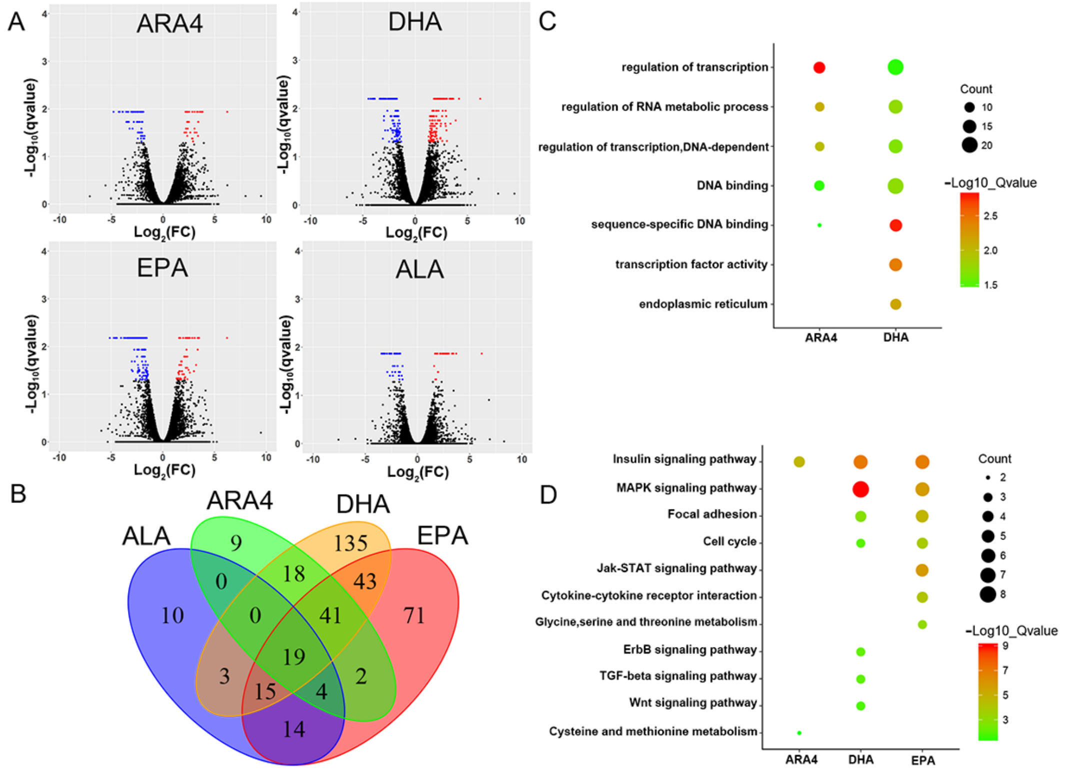


Supplementary Figure 2. Volcano plots and Venn diagram for DEGs in muscle tissues.

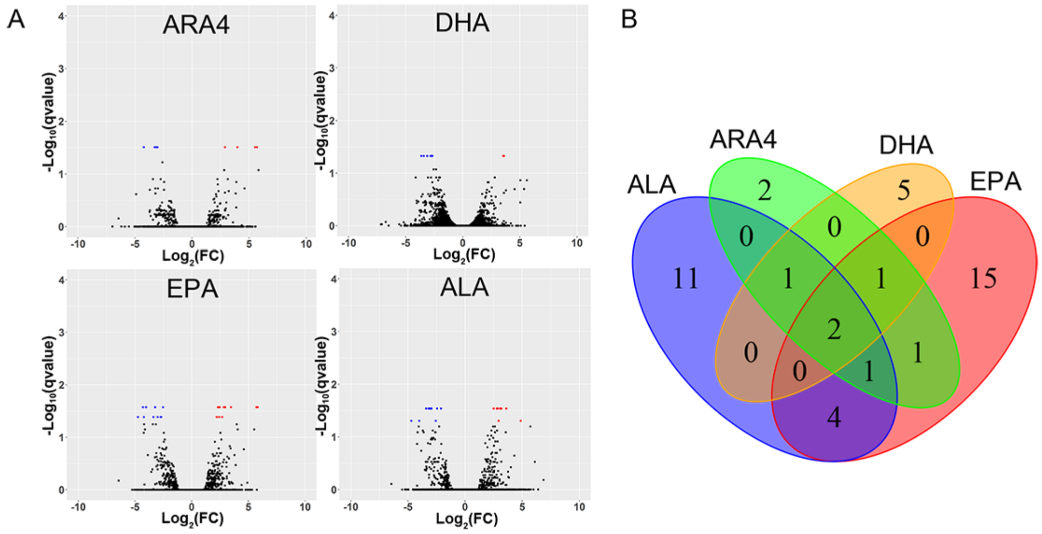

Supplementary Figure 3. Clustering of DEGs in three tissues for four traits.

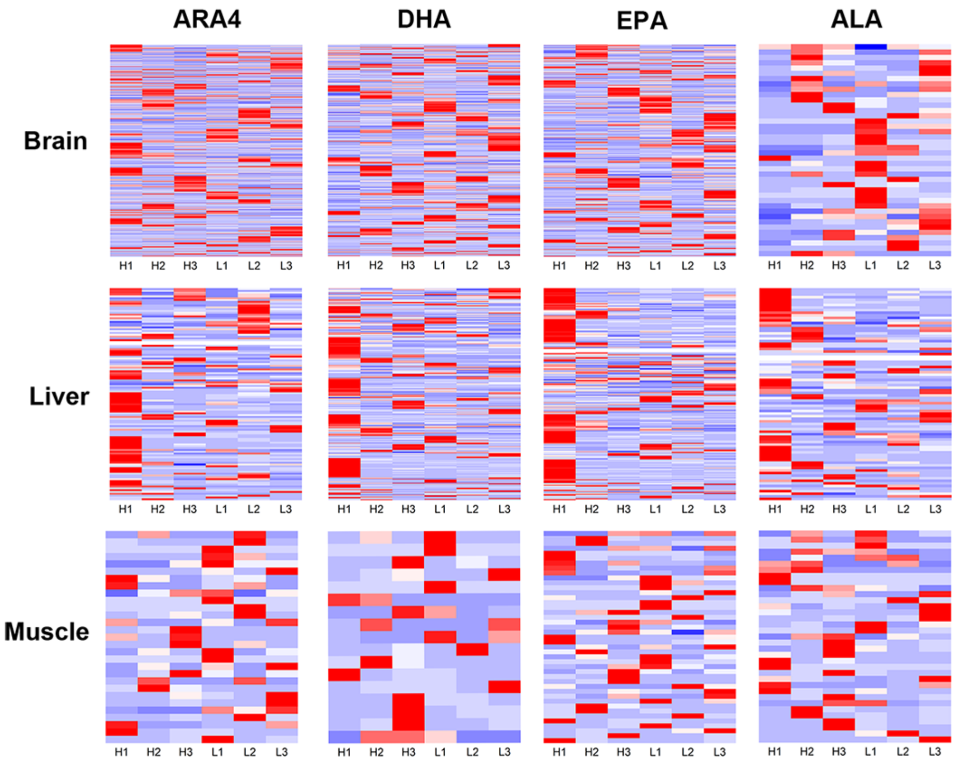

Supplement: Supplementary file 1 [file Image_1.pdf]
